# Toll-like receptors 2 and 4, and bacterial proteins in IgG4-related sialadenitis, other types of chronic sialadenitis and sialolithiasis

**DOI:** 10.1080/20002297.2024.2382633

**Published:** 2024-07-24

**Authors:** Elin Waltimo, Mine Eray, Antti Mäkitie, Caj Haglund, Timo Atula, Jaana Hagström

**Affiliations:** a Department of Oral and Maxillofacial Diseases, University of Helsinki, Helsinki, Finland; b Department of Pathology, University of Helsinki and Helsinki University Hospital, Helsinki, Finland; c Department of Otorhinolaryngology – Head and Neck Surgery, University of Helsinki and Helsinki University Hospital, Helsinki, Finland; d Research Program in Systems Oncology, Faculty of Medicine, Helsinki, Helsinki, Finland; e Research Programmes Unit, Translational Cancer Medicine, University of Helsinki, Helsinki, Finland; f Department of Surgery, University of Helsinki and Helsinki University Hospital, Helsinki, Finland; g Department of Oral Pathology and Radiology, University of Turku, Turku, Finland

**Keywords:** Immunoglobulin G4, head and neck, salivary gland, submandibular gland, *Porphyromonas gingivalis*, lipopolysaccharide

## Abstract

**Background:**

The association of chronic sclerosing sialadenitis and IgG4-related disease (IgG4-RD) has resulted in the more frequent identification of IgG4-positivity in submandibular gland inflammations, also uncovering IgG4 overexpression in nonspecific inflammations. These findings lead us to hypothesise that IgG4-positive sialadenitis represents a continuous inflammatory process overlapping histologically with IgG4-RD, possibly differing in aetiology. However, the antigen underlying IgG4 overexpression in IgG4-positive sialadenitis and IgG4-RD remains unknown.

**Materials and methods:**

Here, we investigated toll-like receptor (TLR) – mediated bacterial inflammation in submandibular gland tissues of patients with IgG4-positive and IgG4-negative chronic inflammatory lesions of the submandibular gland (*n* = 61), with noninflamed submandibular glands serving as controls (*n* = 4). Utilising immunohistochemistry, we assessed the expression of TLR2 and TLR4, lipopolysaccharide (LPS) and the *P. gingivalis*-specific antigen gingipain R1.

**Results:**

We observed TLR2- and TLR4-immunopositivity in 64 (98%) samples. However, TLR2 and TLR4 staining intensity was significantly stronger in the IgG4-positive group. LPS- and gingipain R1 immunopositivity were observed in 56 (86%) and 58 (89%) samples, respectively. LPS-positivity localised exclusively in mast cell-like cells, while gingipain R1-positivity remained scarce.

**Conclusions:**

A stronger TLR2 or TLR4 expression in IgG4-positive sialadenitis may indicate a tissue-related factor underlying this form of chronic sialadenitis. LPS- and *P. gingivalis* immunopositivity remained weak throughout this series. Thus, gram-negative bacteria may not represent pathogens underlying these forms of chronic sialadenitis.

## Introduction

Chronic nonspecific sialadenitis typically has an obstructive origin and, in the submandibular gland, sialolithiasis is often associated with obstructive sialadenitis [1,2]. Other typical causes underlying sialadenitis may include bacterial or viral infections or autoimmune diseases [3,4].

A specific type of submandibular sialadenitis, called chronic sclerosing sialadenitis or Küttner’s tumour, has been the subject of rigorous research since the discovery of a systemic fibroinflammatory disease entity known as immunoglobulin G4-related disease (IgG4-RD) [5–8]. Since its discovery, IgG4-RD has come to encompass many diseases with a previously elusive aetiology, among these chronic sclerosing sialadenitis, widely regarded as the manifestation of IgG4-RD in the submandibular gland [9,10].

While chronic sclerosing sialadenitis has been considered synonymous with IgG4-RD, the histopathological distinction between IgG4-RD and other inflammatory diseases of the submandibular gland does not appear as straightforward as previous research implies. In two of our recent studies, we identified the presence of inflammatory infiltrates overexpressing IgG4-positive plasma cells, not only in tissue samples from patients with chronic sclerosing sialadenitis, but also in tissue samples from glands removed due to nonspecific chronic sialadenitis and sialolithiasis. In fact, in our cohort, chronic sclerosing sialadenitis rarely associated with IgG4 overexpression, with true IgG4-RD not confirmed in any of our patients with chronic sclerosing sialadenitis [11]. Our results led us to hypothesise that an inflammatory entity histologically overlapping with, but likely aetiologically differing from genuine IgG4-RD exists. In this inflammatory continuum, numerous IgG4-positive plasma cells appear in the affected submandibular gland resulting from a prolonged inflammation stemming from an unidentified antigen [11,12]. Thus far, the significance of IgG4 overexpression in submandibular sialadenitis remains unclear. Specifically, it remains unknown whether IgG4 overexpression in submandibular sialadenitis represents a new subtype of sialadenitis or if the abundance of IgG4-positive plasma cells is simply a result of a continuous inflammatory process. Perhaps, histopathological features typical for chronic sclerosing sialadenitis or even IgG4-RD develop following prolonged inflammation.

Toll-like receptors (TLRs) are a family of transmembrane proteins that activate innate immune responses through recognition of pathogen-associated molecular patterns (PAMPs) and damage-associated molecular patterns (DAMPs). In total, 10 human TLRs have been identified, whereby each TLR can trigger an immune response through the recognition of several different ligands [13,14]. TLR2 and TLR4 are cell surface TLRs which recognise microbial membrane components such as bacterial lipopolysaccharides (LPS) and peptidoglycans. Specifically, TLR2 recognises peptidoglycan and lipoprotein components of a variety of microbial pathogens, and TLR4 primarily binds to LPS expressed by gram-negative bacteria [15,16]. In addition to pathogen recognition, TLR-mediated inflammatory pathways are also activated through DAMPs, which consist of endogenous molecules released during tissue damage or apoptosis. Both TLR2 and TLR4 recognise a plethora of DAMPs and act as mediators in many inflammatory responses activated by tissue damage. DAMP-mediated TLR responses have been implied in many inflammatory and autoimmune diseases, where excessive TLR signalling leads to an unrestricted inflammatory response [17–19].

Bacterial sialadenitis is often a polymicrobial infection caused by several bacteria residing in the oral cavity. Bacteria frequently associated with submandibular sialadenitis include, for instance, *Staphylococcus aureus* (*S. aureus*), *Streptococcus viridans* (*S. viridans*), *Haemophilus influenzae* (*H. influenzae*), *Enterobacteriaceae spp.* and anaerobes such as *Prevotella, Fusobacterium spp.* and *Peptostreptococcus* [3,4]. Many of the bacteria found in sialadenitis lesions are involved in the pathogenesis of other oral diseases, namely dental caries and periodontitis [20–22]. One of the major pathogens responsible for the development of periodontitis is the gram-negative anaerobe *Porphyromonas gingivalis* (*P. gingivalis*) [23]. In recent years, *P. gingivalis* has piqued the interest of researchers given its links to an increasing number of systemic diseases, including cardiovascular disease, Alzheimer’s disease and rheumatoid arthritis [23,24]. *P. gingivalis* expresses a unique set of virulence factors called gingipains, a family of proteinases that allow *P. gingivalis* to modify innate immune and inflammatory responses. One proposition hypothesises that gingipains are responsible for the involvement of *P. gingivalis* in diseases beyond the oral cavity [24,25]. Furthermore, *P. gingivalis* is known to activate inflammatory responses through both TLR2- and TLR4-mediated pathways, another mechanism thought to aid in the systemic involvement of *P. gingivalis* [26,27].

The causes of IgG4 overexpression in chronic sialadenitis remain unknown. Furthermore, a potential bacterial aetiology as a cause of specific forms of chronic sialadenitis such as chronic sclerosing sialadenitis has not been widely examined. In addition, IgG4-RD is strongly implied as an autoimmune disease, although the triggering antigens behind this disease are yet to be identified. Therefore, this study aimed to investigate the expression of TLRs recognising bacterial PAMPs, and the bacterial virulence factors LPS and gingipain R1 in various types of chronic sialadenitis, with special emphasis placed on IgG4-overexpressing sialadenitis. We also aimed to examine the possible background of bacterial inflammation mediated through TLRs in these diseases. To do so, we utilised immunohistochemical methods to assess the expression of antigens for TLR2, TLR4, LPS and gingipain R1. By analysing the expression patterns of these antigens, we attempt to gain knowledge regarding the causative agents for and the possible association between chronic sialadenitis and the newly discovered IgG4-overexpressing sialadenitis, including true IgG4-RD.

## Materials and methods

### Ethics

The local research ethics committee approved the study design (HUS 967/2017, record number: 192/13/03/02/16), and an updated institutional study permission was granted (§45/2022).

### Cohort and patients

**Figure 1.**
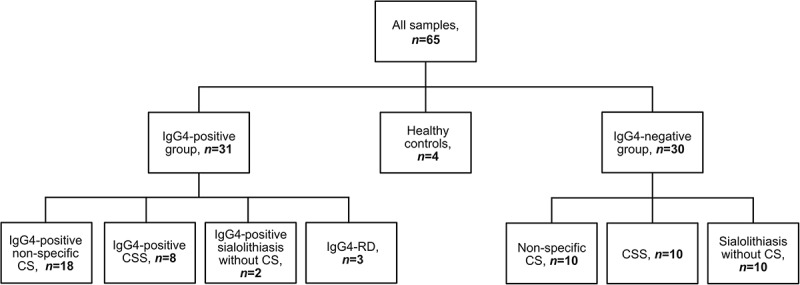
Samples were divided into the following study groups and subgroups.

Abbreviations: IgG4-positive, ≥70 IgG4-positive plasma cells in a high-power field of x400 magnification. CS, chronic sialadenitis; CSS, chronic sclerosing sialadenitis; IgG4-RD, IgG4-related disease.

Patients in this study were selected from cohorts formed from two previous retrospective studies, where we examined the association of IgG4-RD and IgG4-positive plasma cell infiltrates in patients diagnosed with chronic sclerosing sialadenitis and patients diagnosed with nonspecific chronic sialadenitis and sialolithiasis [11,12]. From these cohorts, we included 61 samples in total for this current series, from which we formed two study groups: an IgG4-positive group and an IgG4-negative group. The IgG4-positive group comprised of all samples (*n* = 31) classified as IgG4-positive (≥70 IgG4-positive plasma cells in a high-power field of x400 magnification) or as true IgG4-RD in our two previous studies [11,12]. In the IgG4-negative group, we included 30 consecutively selected samples from patients that did not exhibit IgG4 positivity. The samples in the IgG4-positive group and the IgG4-negative group were further divided into subgroups based on their originally assigned histological diagnosis. The division of patients into study groups and further into subgroups is described in detail in Figure 1. Tissue samples from healthy submandibular glands (*n* = 4) removed during neck dissection served as control samples. We collected data on sex and age at the time of submandibular gland surgery from patient records for all patients except for controls. In the study groups, 32 (52%) patients were male and 29 (48%) were female, the median age of the patients at the time of surgery was 57 in the IgG4-positive group (range 25–86) and 52 (range 19–79) in the IgG4-negative group. Table 1 summarises the clinical characteristics of the patients across subgroups.

**Table 1.** Clinical characteristics of patients in the IgG4-positive and IgG4-negative groups.

*IgG4-positive samples*	Sex (M/F)	Age, range	Age, mean	*IgG4-negative samples*	Sex (M/F)	Age, range	Age, mean
Nonspecific sialadenitis with or without sialolithiasis (*n* = 18)	11/7	27–86	54.7	Nonspecific sialadenitis with or without sialolithiasis (*n* = 10)	3/7	35–79	51.1
Chronic sclerosing sialadenitis (*n* = 8)	5/3	29–79	57.1	Chronic sclerosing sialadenitis (*n* = 10)	6/4	38–79	60.0
Sialolithiasis without sialadenitis (*n* = 2)	0/2	55–56	55.5	Sialolithiasis without sialadenitis (*n* = 10)	6/4	19–61	43.7
IgG4-related disease (*n* = 3)	1/2	25–63	49.3				

Abbreviations: M, male; F, female; IgG4-positive, ≥70 IgG4-positive plasma cells in a high-power field of x400 magnification.

### Immunohistochemical staining

The formalin-fixed, paraffin-embedded submandibular gland samples were cut into 4-µm-thick sections and mounted onto glass slides. Immunohistochemical staining for TLR2, TLR4, LPS, and gingipain R1 was carried out according to the following protocol. For TLR2 staining, we used a rabbit polyclonal antihuman TLR2 antibody (NB100–56720, Novus Biologicals); for TLR4 staining, we used a mouse monoclonal antihuman TLR4 antibody (sc -293,072, Santa Cruz Biotechnology, Inc.); for LPS staining, we used a mouse monoclonal anti-*E. coli* LPS antibody (Abcam 35,654, Abcam); and for *P. gingivalis* gingipain R1 staining, we used a rabbit polyclonal anti-*P. gingivalis* gingipain R1 antibody (biorbyt orb243611, Biorybt). Deparaffinisation and antigen retrieval for all samples was performed in a pretreatment module (Agilent Dako, CA, USA) using a pH 9 retrieval solution (EnVision Flex target retrieval solution, high pH, DM828) for 15 min at 98°C. The sections were then stained in an Autostainer 480S (LabVision) using the EnVision Flex Detection System (Agilent Dako, CA, USA). Samples were treated with an EnVision Flex peroxidase-blocking reagent (SM801) for 15 min. Slides were incubated overnight with a rabbit polyclonal TLR2 antibody (1:300), mouse monoclonal anti-*E. coli* LPS antibody (1:100), and rabbit polyclonal *P. gingivalis* gingipain R1 antibody (1:600). The mouse monoclonal TLR4 antibody (1:1500) slides were incubated for 1 h. Appropriate dilution and incubation time was optimised for each antibody, and a Dako REAL antibody diluent was used for antibody dilution. Subsequently, slides stained for TLR2, LPS and gingipain R1 underwent 20-min incubation with a peroxidase-conjugated EnVision Flex/HRP (SM802) rabbit/mouse (ENV) reagent. Slides stained for TLR4 underwent incubation with a secondary peroxidase-conjugated EnVision Flex/HRP (SM802) rabbit/mouse antibody (ENV) for 30 min. Slides stained for TLR2 and TLR4 were visualised using DAB chromogen (EnVision Flex DAB, DM827) for 10 min, and slides stained for LPS and gingipain R1 were visualised using a Romulin AEC chromogen kit (Biocare Medical, Romulin AEC chromogen kit, RAEC810). Mayers haematoxylin (S3309, Dako) was used for counterstaining.

### Scoring of immunohistochemical staining

Tissue samples were scored by two experienced pathologists (JH and ME) blinded to sample data. When scoring the TLR2 and TLR4 staining, each sample was assessed for the staining intensity of three items: the lymphoplasmacytic infiltrates, the ductal epithelium and the acini. In each sample, each item was given a score ranging from 0 to 3: 0 indicating negative, 1 indicating mild staining, 2 moderate staining and 3 strong staining. Samples stained for LPS and gingipain R1 were given a score of either 0 for negative or 1 for positive.

### Statistical analyses

All statistical analyses were performed using IBM SPSS statistics (version 28.0.0.0). The nonparametric Mann Whitney U-test was utilised to analyse differences in the staining intensity of TLR2 and TLR4 and differences in the frequency of LPS and gingipain R1 staining positivity between the IgG4-positive and IgG4-negative groups. The control group was not included in the statistical analyses due to the small sample size. We considered *p* < 0.005 as statistically significant.

## Results

### TLR2

**Figure 2.**
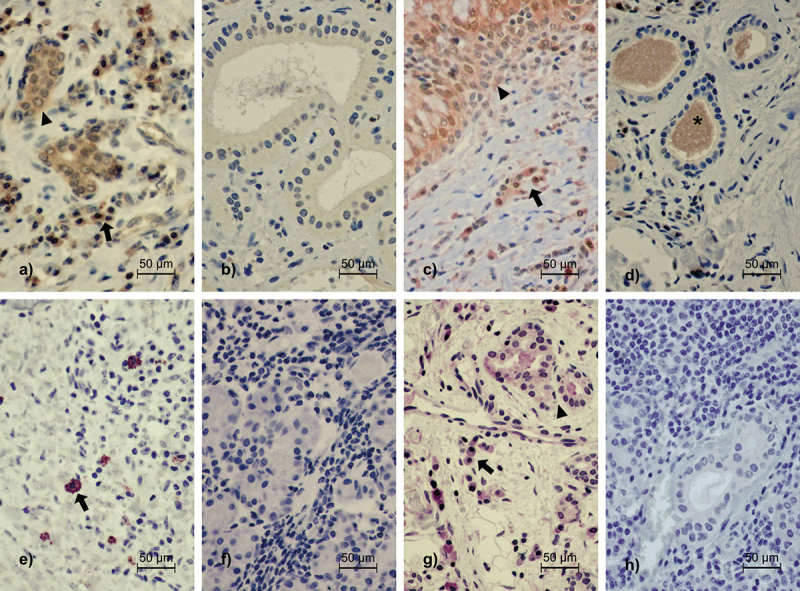
Staining patterns of TLR2, TLR4, gingipain R1 and LPS. (a) TLR2-positive sample from a patient with IgG4-positive chronic sclerosing sialadenitis. Intense staining is observed in the plasma cells (arrow) and the epithelial cells of the striate ducts (arrowhead). (b) TLR2-negative sample from a patient with IgG4-negative nonspecific chronic sialadenitis. (c) TLR4-positive sample from a patient with IgG4-positive sialadenitis. Intense positivity is observed in the plasma cells interspersing the fibrotic tissue (arrow) and the epithelial cells of the interlobular duct (arrowhead). (d) Negative TLR4 staining in a patient with IgG4-negative chronic sclerosing sialadenitis. Only the excretory material within the striate ducts is stained with TLR4 (asterisk). (e) Positive LPS staining in a patient with IgG4-positive chronic sclerosing sialadenitis. Staining positivity is seen exclusively in the mast cell-like cells (arrow). (f) Negative LPS staining in a patient with IgG4-RD. (g) Positive gingipain R1 staining in a patient with IgG4-positive chronic sclerosing sialadenitis. Weak staining positivity is present in inflammatory cells (arrow) and the epithelium of striate ducts (arrowhead). (h) Negative gingipain R1 staining in a patient with IgG4-RD. All photographs are taken at x400 magnification.

Abbreviations: TLR, toll-like receptor; LPS, lipopolysaccharide; IgG4-positive, ≥70 IgG4-positive plasma cells in a high-power field of x400 magnification; IgG4-RD, IgG4-related disease.

**Figure 3.**
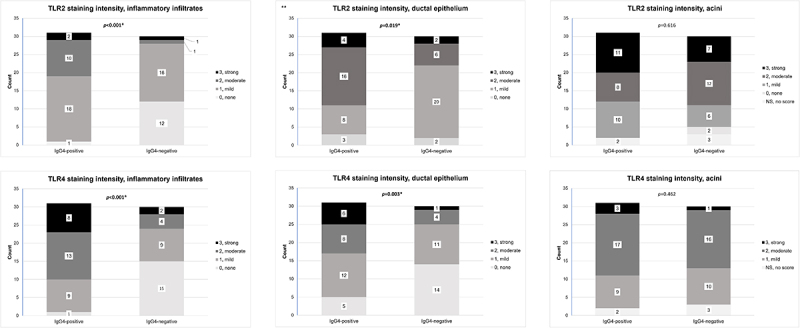
Staining scores for TLR2 and TLR4 were individually assessed in each sample for the inflammatory infiltrates, ducts and acini. The Mann Whitney U-test was used to assess differences in the distribution of scores between the IgG4-positive and the IgG4-negative groups. The significance level was set at *p* < 0.05 and statistically significant comparisons are noted with an asterisk (*).

Abbreviations: IgG4-positive, ≥70 IgG4-positive plasma cells in a high-power field of x400 magnification; TLR, toll-like receptor.

All samples except one belonging to the IgG4-negative nonspecific sialadenitis group exhibited varying degrees of TLR2-positive staining, ranging from mild to strong in one or multiple evaluated structures. These structures included lymphoplasmacytic infiltrates, ductal epithelium and acini (Table 2). The staining patterns observed in TLR2-positive samples appear in Figure 2. We observed a strong TLR2 expression most frequently in the acini, in both the IgG4-positive and the IgG4-negative groups. In comparison, TLR2 positivity of the lymphoplasmacytic infiltrates was significantly more frequent and intense in the IgG4-positive group than the IgG4-negative group (*p* < 0.001). Moreover, in the IgG4-positive group, the ductal epithelium expressed TLR2 more intensely compared to the IgG4-negative group (*p* = 0.019) (Figure 3). The distribution of positive staining scores in the acini did not statistically differ between the IgG4-positive and the IgG4-negative groups (*p* = 0.616). All samples in the control group showed mild-to-moderate TLR2 positivity in the lymphoplasmacytic infiltrates and acini. In the ductal epithelium, one control sample exhibited a strong TLR2 expression, with another three exhibiting TLR2 staining positivity of the ductal epithelium as mild or moderate (Table 2).

**Table 2.** Distribution of TLR2 and TLR4 staining positivity in evaluated structures comparing IgG4-positive, IgG4-negative and control groups. Evaluated structures include the lymphoplasmacytic infiltrates, ductal epithelium and acini.

TLR2										
**Negative score, 0; positive score, 1–*3*	*Lymphoplasmacytic infiltrate*			*Ductal epithelium*			*Acini*			
	Negative	Positive	Statistical comparison †	Negative	Positive		Negative	Positive	ND***	Statistical comparison †
IgG4-positive group, all *n* = 31*	1 (3%)	30 (97%)	***p* < 0.001**	3 (10%)	28 (90%)	***p***** = 0.019**	0 (0%)	29 (93%)	2 (7%)	*p* = 0.616
*IgG4-positive nonspecific sialadenitis with or without sialolith, n = 18*	0	18		0	18		0	18	0	
*IgG4-positive chronic sclerosing sialadenitis, n = 8*	1	7		2	6		0	7	1	
*IgG4-positive sialolithiasis without sialadenitis, n = 2*	0	2		0	2		0	2	0	
*IgG4-RD, n = 3*	0	2		1	2		0	2	1	
IgG4-negative group, all *n* = 30*	16 (53%)	14 (47%)	***p*****< 0.001**	2 (7%)	28 (93%)	***p***** = 0.019**	2 (7%)	25 (83%)	3 (10%)	*p* = 0.616
*Nonspecific sialadenitis, n = 10*	7	3		1	9		1	9	0	
*Chronic sclerosing sialadenitis, n = 10*	2	8		0	10		1	6	3	
*Sialolithiasis without sialadenitis, n = 10*	7	3		1	9		0	10	0	
**Control group, all *n* = 4**	0 (0%)	4 (100%)		0 (0%)	4 (100%)		0 (0%)	4 (100%)	0 (0%)	
TLR 4										
IgG4-positive group, all *n* = 31*	1 (3%)	30 (97%)	***p*****< 0.001**	**5 (16%)**	**26 (84%)**	***p***** = 0.003**	0 (0%)	29 (94%)	2 (6%)	*p* = 0.462
*IgG4-positive nonspecific sialadenitis with or without sialolith, n = 18*	1	17		2	16		0	18	0	
*IgG4-positive chronic sclerosing sialadenitis, n = 8*	0	8		0	8		0	7	1	
*IgG4-positive sialolithiasis without sialadenitis, n = 2*	0	2		2	0		0	2	0	
*IgG4-RD, n = 3*	0	3		1	2		0	2	1	
IgG4-negative group, all *n* = 30*	15 (50%)	15 (50%)	***p*****< 0.001**	14 (47%)	16 (53%)	***p***** = 0.003**	0	27 (90%)	3	*p* = 0.462
*Nonspecific sialadenitis, n = 10*	6	4		7	3		0	10	0	
*Chronic sclerosing sialadenitis, n = 10*	2	8		3	7		0	7	3	
*Sialolithiasis without sialadenitis, n = 10*	7	3		4	6		0	10	0	
Control group, all *n* = 4*	2 (50%)	2 (50%)		0 (0%)	4 (100%)		1 (25%)	3 (75%)	0 (0%)	

*Due to the small patient numbers in each subgroup prevalence percentages are given only to ‘all’, where *n* = 31 in the IgG4-positive group, *n* = 30 in the IgG4-negative group, and *n* = 4 in the control group;**0, negative; 1, mild staining; 2, moderate staining; 3, strong staining; ***ND, no data; † Statistical comparisons of the TLR2 and TLR4 staining patterns were performed only between the IgG4-positive and the IgG4-negative groups, the control group was not included in the statistical comparisons due to the small sample size. The non-parametric Mann-Whitney U test was used for analysis and the significance level was set at *p* < 0.05, significant p-values appear in bolded text.

### TLR4

All samples except one belonging to the IgG4-negative chronic sclerosing sialadenitis group showed TLR4 positivity ranging from mild to strong in one or multiple evaluated structures. These structures included lymphoplasmacytic infiltrates, ductal epithelium and acini (Table 2). A stronger staining intensity was generally more frequent in lymphoplasmacytic infiltrates, ductal epithelium, and acini in the IgG4-positive group. Furthermore, TLR4 expression in the lymphoplasmacytic infiltrates and the ductal epithelium was significantly more intense, and more frequent in the IgG4-positive group compared to the IgG4-negative group (*p* < 0.001 and *p* = 0.003, respectively). The intensity of the TLR4 staining in the acini did not differ significantly between the IgG4-positive and the IgG4-negative groups (*p* = 0.462) (Figure 3). Figure 2 illustrates the observed staining patterns for TLR4. All the samples in the control group showed mild TLR4 positivity in the ductal epithelium. In three of the control samples, mild positivity was also observed in the acini. Lastly, in two of the control samples, mild and moderate TLR4 positivity was also observed in the lymphoplasmacytic infiltrates (Table 2).

In total, five tissue samples exhibited advanced fibrosis and severe acinar atrophy, such that no acini were present to score for TLR2 and TLR4. The lymphoplasmacytic infiltrates and ductal epithelium of these samples were, however, normally scored for TLR2 and TLR4 staining intensity (Table 2). Of the five samples with severe acinar atrophy, one fell into the IgG4-RD group, one into the IgG4-positive chronic sclerosing sialadenitis group and three in the IgG4-negative chronic sclerosing sialadenitis group.

### LPS

**Figure 4.**
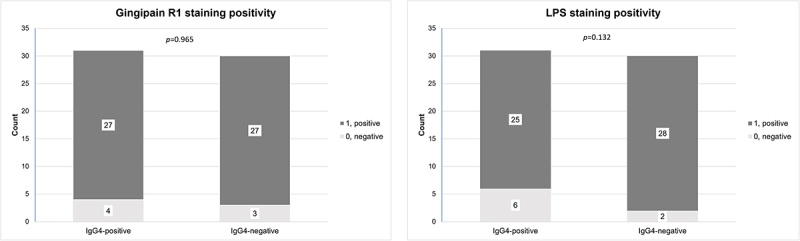
Distribution of gingipain R1 and LPS positivity between the IgG4-positive and the IgG4-negative groups. The Mann Whitney U-test was used to assess differences in the distribution of staining positivity between the IgG4-positive and the IgG4-negative groups. The significance level was set at *p* < 0.05.

Abbreviations: IgG4-positive, ≥70 IgG4-positive plasma cells in a high-power field of x400 magnification; LPS, lipopolysaccharide.

In total, 56 (88%) samples stained positive for LPS. In all of these samples, staining positivity was exclusively observed in mast cell-like cells, scarcely scattered throughout the glandular tissues (Figure 2). Altogether, eight samples (13%) were negative for LPS, among which the three patients with IgG4-RD were included. The frequency of LPS positivity showed no difference between IgG4-positive and IgG4-negative groups (*p* = 0.132) (Figure 4). All samples in the control group stained positive for LPS.

### gingipain R1

**Figure 5.**
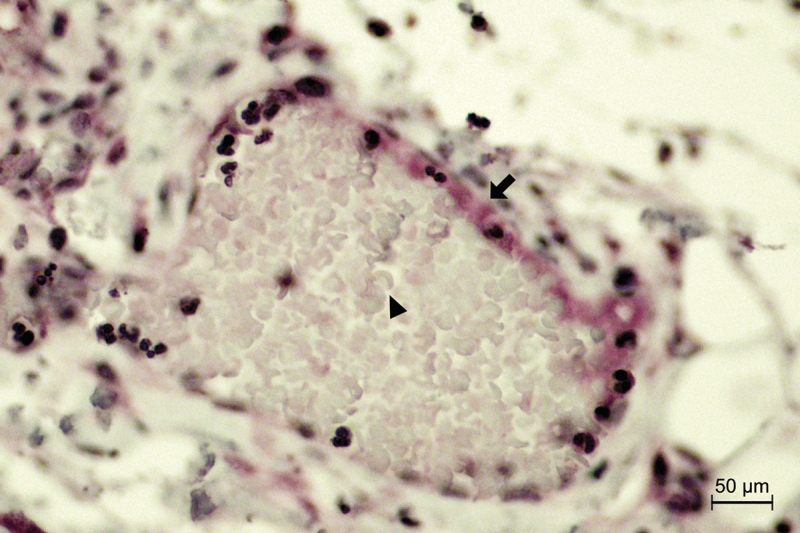
Gingipain R1 immunopositivity in the blood vessel endothelium (arrow) in a healthy control. Erythrocytes are seen inside the vessel (arrowhead). x400 magnification.

Upon gingipain R1 staining, we observed positivity in 58 (89%) samples. Weak positive staining for gingipain R1 was most frequently detectable at local foci, typically located on the periphery of the tissue sample. Staining positivity of a low intensity was predominantly detected in the secretory granules of the acini, in the epithelial walls of the striated ducts and in the inflammatory cells (Figure 2). In 20 (31%) samples, we observed positive staining in the endothelial walls of the blood vessels. The vessels with positive staining were located in the capsule surrounding the salivary gland parenchyma (Figure 4). The staining intensity for gingipain R1 tended to be more intense towards the periphery of the tissue sample, while the core of the sample remained mostly negative. We detected no significant difference in gingipain R1 staining positivity when the IgG4-positive and the IgG4-negative groups were compared (*p* = 0.965) (Figure 5). All samples in the control group showed a positive gingipain R1 expression.

## Discussion

In this series, we observed a significantly stronger immunopositivity of TLR2 and TLR4 in the IgG4-positive group, although a weaker positivity was also observed in the IgG4-negative group and in the controls. The observed TLR expression does not appear to be related to infections caused by gram-negative bacteria, given that the staining positivity for LPS and gingipain R1 was only scarcely located and did not co-localise with the TLR2 and TLR4 staining patterns. Moreover, the expression pattern of the bacterial virulence factors did not differ between the IgG4-positive and the IgG4-negative group. Thus, a bacterial antigen does not appear to cause IgG4 overexpression in the submandibular gland. The observed expression patterns of TLR2 and TLR4 may indicate that tissue damage-related factor rather than a bacterial origin underlies the TLR2 and TLR4 activation observed in both the IgG4-positive and IgG4-negative groups.

To obtain detailed observations on how TLRs are expressed in the submandibular gland, we scored the intensity of TLR2 and TLR4 staining in three different structures: the lymphoplasmacytic infiltrates, the ductal epithelium, and the acini. As TLRs are expressed on a wide variety of cells, including immune cells and epithelial cells, the ductal epithelium, and inflammatory infiltrates represented cell structures where TLRs were likely to be expressed [28]. Moreover, as one aim of our study was to examine the differences in TLR expression between IgG4-overexpressing and nonspecific sialadenitis, the inflammatory cell infiltrates were a key target for assessing differences in TLR immunoexpression.

In addition to recognising ligands from a variety of bacteria, TLR2 and TLR4 mediate inflammatory responses through recognition of many endogenous ligands produced following tissue damage [19]. Under normal circumstances, these DAMP-activated inflammatory responses aim to initiate tissue repair. For instance, TLR4-mediated autophagy has been proposed as a protective response to the tissue damage caused by chronic inflammation in the salivary gland [29]. Thus, the demonstrated immunopositivity for TLR2 and TLR4 could be indicative of TLR activation by tissue damage – or necrosis-related molecules released following tissue destruction caused by prolonged inflammation. However, DAMP-mediated TLR activation as a response to chronic nonspecific inflammation may not fully explain the distinctly stronger expression of TLR2 and TLR4 we observed in the IgG4-positive group.

When regulation of TLR signalling fails, it can cause the signal transduction to become aberrant, thereby leading to an excessive inflammatory response [19]. Excessive TLR signalling, caused by the overexpression of endogenous TLR ligands, contributes to many inflammatory and autoimmune diseases [18,19]. For instance, Shimizu et al. examined autoimmune sialadenitis in mouse models, demonstrating that the overexpression of TLR1–4 and TLR9 might be involved in the development or progression of sialadenitis in their models, further theorising that some types of autoimmune sialadenitis can be TLR mediated [30]. Thus, the stronger expression patterns of TLR2 and TLR4 we observed in the IgG4-positive group may indicate an aberrant role of TLR-mediated inflammatory responses in IgG4-overexpressing sialadenitis. Moreover, mechanisms involving crosstalk between innate and acquired immunity, in which TLR signalling acts as a connecting link, are implied in the immunopathogenesis of IgG4-RD [31]. For instance, findings by Ishiguro et al. suggest a TLR-mediated pathological mechanism causing tissue fibrosis in patients with IgG4-RD of the salivary glands. More specifically, Ishiguro et al. found that M2 macrophages activated by TLR7 produced profibrotic cytokines in patients with IgG4-RD [32]. Other studies have also indicated that cytokines produced by antigen-presenting cells act as a T-cell independent mechanism driving the IgG4 class switch in IgG4-RD [31,33,34]. Furthermore, in these studies, the expression patterns of TLRs and antigen-presenting cells and their secreted cytokines appeared localised around ectopic germinal centres and in lymphocytic infiltrates in patients with IgG4-RD [32]. These results agree with our findings, whereby both TLR2 and TLR4 expressions were significantly stronger in the lymphoplasmacytic infiltrates of samples in the IgG4-positive group. Taken together, these findings support the idea that innate immune responses mediated by TLR2 and/or TLR4 signalling play a role in the overexpression of IgG4-positive plasma cells in IgG4-positive sialadenitis, perhaps through mechanisms similar to those described in true IgG4-RD. However, further studies targeting the cells and signalling molecules of innate immunity are warranted in order to gain additional insight into the significance of the TLR2 and TLR4 expression patterns in IgG4-positive forms of chronic sialadenitis.

Although we observed positive staining patterns for LPS and gingipain R1 in 88% and 89% of our samples, respectively, such patterns were often restricted to single cells or localised within intact salivary gland structures. It is, thus, likely that our findings merely demonstrate that *P. gingivalis* and other gram-negative bacteria can reside in the submandibular gland without activating notable immune response. The presence of bacteria in these chronic inflammatory diseases is possibly explained by LPS-expressing gram-negative bacteria, including *P. gingivalis*, entering the submandibular gland through Wharton’s duct when salivary flow is restricted [2,35,36]. Furthermore, in 20 (31%) samples, positivity for gingipain R1 could be observed in the endothelial walls of the blood vessels, possibly implying that the blood vessels serve as an alternative route via which *P. gingivalis* enters the glandular tissues. LPS and gingipain R1 positivity was found in all our control samples as well. Due to the submandibular glands’ close anatomical relationship to the oral cavity, which hosts a rich microbiome, it may not be totally unexpected to find microbial antigens in our control specimens. Many gram-negative bacteria, including *P. Gingivalis* are opportunistic pathogens which are particularly prevalent in a dysbiotic oral flora, furthermore oral dysbiosis may cause ectopic colonisation of oral pathogens in tissues outside the oral cavity [37]. Thus, oral dysbiosis could possibly act as a factor causing bacteria to descend the submandibular gland duct even in the absence of preceding sialadenitis.

We did not assess the expression of surface proteins of the gram-positive bacteria in our cohort. Since TLR2 recognises ligands from a wide range of gram-positive bacteria [15], we cannot based on these results fully exclude a bacterial antigen as an alternative TLR2-activating ligand as opposed to DAMP-mediated TLR2 overexpression. Thus, screening for the TLR ligands of gram-positive bacteria is warranted in order to definitively identify the role of bacteria in these chronic inflammatory diseases of the submandibular gland. An additional limitation of this study is the small size of the control group. The differences between the sample sizes of the study groups (*n* = 31 and *n* = 30) and the control group (*n* = 4) did not allow for trustworthy statistical comparisons between the IgG4-positive and IgG4-negative groups and the controls. Thus, a larger control group would be needed to establish whether TLR2 and TLR4 are constitutionally expressed in healthy submandibular glands, which might be suggested by our findings of TLR2 and TLR4 positivity in all controls. Similarly, a larger sample of healthy controls could shed light on whether the presence of antigens from gram-negative bacteria is ubiquitous in healthy salivary glands.

## Conclusions

A strong expression of TLR2 and TLR4 in IgG4-positive forms of sialadenitis may indicate a tissue-related factor as an antigen in this specific form of chronic sialadenitis. Immunopositivity of gram-negative bacteria remained weak throughout this patient series, such that neither *P. gingivalis* nor other LPS-expressing gram-negative bacteria appeared to serve as pathogens underlying different forms of chronic sialadenitis. However, the involvement of gram-positive bacteria in these diseases cannot be excluded based on these results.
